# Unifying spatiotemporal and frequential attention for traffic prediction

**DOI:** 10.1038/s41598-024-82759-z

**Published:** 2025-01-06

**Authors:** Qi Guo, Qi Tan, Jun Tang, Benyun Shi

**Affiliations:** 1 College of Computer and Information Engineering, Nanjing Tech University, Nanjing, Jiangsu China; 2 College of Artificial Intelligence, Nanjing Tech University, Nanjing, China

**Keywords:** Traffic flow prediction, Graph neural networks, Attention mechanism, Temporal-frequential attention, Civil engineering, Engineering, Electrical and electronic engineering

## Abstract

Intelligent transportation systems heavily rely on forecasting urban traffic flow, and a variety of approaches have been developed for this purpose. However, most current methods focus on exploring spatial and temporal dependencies in historical traffic data, while often overlooking the inherent spectral characteristics hidden in traffic time series. In this paper, we introduce an approach to analyzing traffic flow in the frequency domain. By integrating attention mechanisms, we comprehensively capture the hidden correlations among space, time, and frequency dimensions. By leveraging deep learning to capture spatial correlations in traffic flow and applying spectral analysis to fuse time series data with underlying periodic correlations in both the time and frequency domains, we develop an innovative traffic prediction model called the Space-Time-Frequency Attention Network (STFAN). The core of this network lies in the application of attention mechanisms, which project the hidden states of current traffic features across the space, time, and frequency domains onto future hidden states. This approach enables a comprehensive learning of the relationships between each dimension and the future states, ultimately allowing for accurate predictions of future traffic flow. We carry out experiments on two publicly available datasets from the California Department of Transportation, PeMS04 and PeMS08, to assess the performance of the proposed model. The results demonstrate that the proposed model outperforms existing baseline models in terms of predictive accuracy, particularly for mid- and long-term traffic flow forecasting. Finally, the ablation study confirmed that the frequency domain characteristics of traffic flow significantly influence future traffic conditions, demonstrating the practical effectiveness of the model.

## Introduction

The advent of Intelligent Transportation Systems (ITS) has transformed urban traffic management through the integration of data analytics^1,2^. Traffic prediction plays a critical role in ITS, as accurate and timely forecasts not only improve road utilization efficiency, alleviate congestion, and reduce accident rates, but also provide crucial data for urban planning and transportation policy development^3^. Despite its importance, traffic forecasting faces numerous challenges, particularly in utilizing historical traffic flow data to uncover spatiotemporal dependencies among network nodes. These challenges have driven ongoing advancements in traffic forecasting research^4,5^.

In the field of traffic forecasting research, two principal methodologies have emerged: model-driven and data-driven approaches^6^. Model-driven methods include a variety of models, such as queuing theory, traffic flow dynamics, and microscopic fundamental diagrams^7,8^. Although these approaches offer distinct advantages in analyzing the static and dynamic attributes of traffic networks, they face limitations when modeling the effects of stochastic variables, such as adverse weather conditions and traffic accidents^5,9^. Furthermore, their effectiveness can be constrained by factors such as the placement of sampling points and the sampling frequency^10^. As a result, model-driven approaches may struggle to accurately predict traffic flow when addressing the complex spatiotemporal dependencies among nodes in a traffic network^11^.

Compared to traditional model-driven approaches, data-driven methods offer significant advantages in traffic flow analysis by fully leveraging vast amounts of traffic data to provide a more comprehensive and effective understanding of traffic patterns^12–14^. In recent years, data-driven approaches have gained widespread use in machine learning-based traffic analysis, prompting the development of various neural network models to explore the spatiotemporal dependencies in traffic data. For example, Recurrent Neural Networks (RNNs) are widely recognized for effectively modeling both short-term and long-term dependencies in time series data^15,16^. In addition, Graph Neural Networks (GNNs) have demonstrated efficiency in analyzing spatial connections within intricate network structures^13,14^. With the emergence of the attention mechanisms and their widespread application across diverse domains, traffic flow forecasting has also reaped benefits from the model’s self-attention mechanism, which is adept at handling interdependencies within the input sequence^17–19^. For example, with the advent of the Transformer model, researchers have used a multi-layer encoder-decoder framework to model the dependencies between traffic sequences^20^. With the combination of graph neural networks, the complex dynamic spatiotemporal dependencies between time and space are effectively learned.

**Fig. 1:**
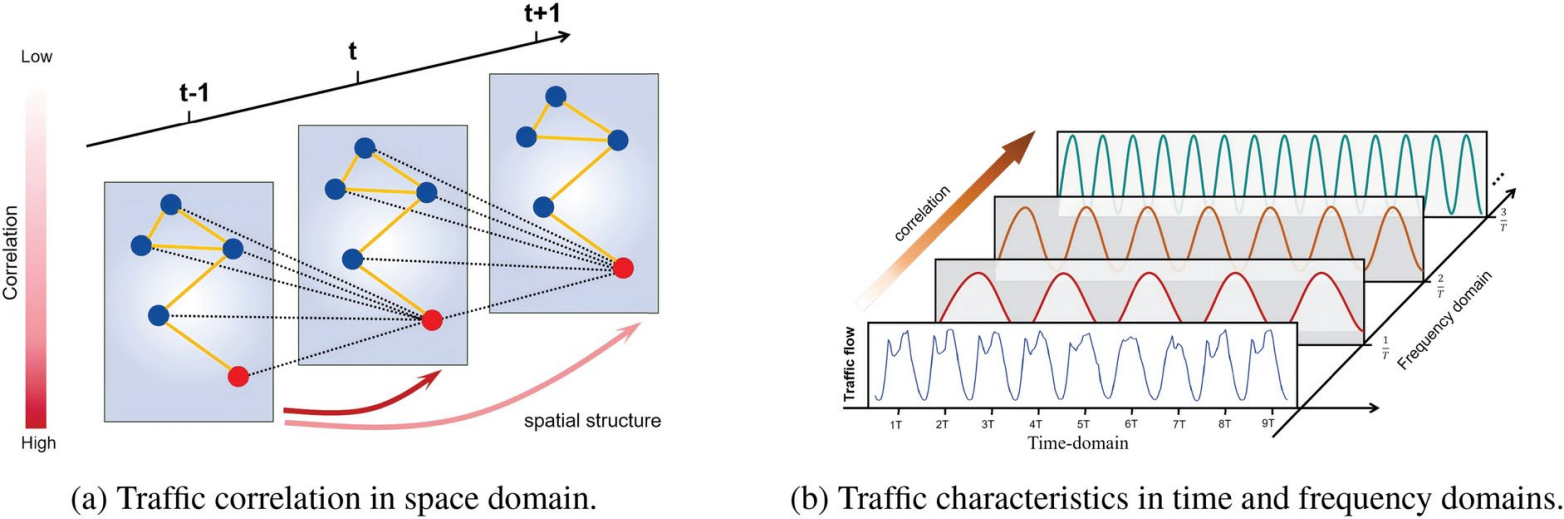
Traffic correlations in the space, time, and frequency domains. (**a**) Correlation of traffic flows in the space domain, where each node has an impact on the nearby nodes at the next moment. (**b**) Traffic characteristics in the time and frequency domains, where a hidden correlation can be observed between the time and frequency domains.

In the field of traffic flow prediction, capturing spatiotemporal dependencies remains a critical challenge. From the perspective of spectral theory^21^, traffic time series data exist not only in the time domain but can also be effectively represented in the frequency domain. Previous studies have largely concentrated on the impact of relationships between consecutive time points in the time domain; however, it is also vital to recognize that characteristics in the frequency domain, such as amplitude, phase variations, and periodicity, are also essential for the accuracy of the prediction. These frequency-domain characteristics present a new method, linking them to spatial and temporal dependencies, which is of notable importance. Spatial characteristics are essential for the prediction of traffic flow due to the complexity of urban road networks^18,22^, where traffic in one area is significantly influenced by the conditions of neighboring areas, as illustrated in Fig. 1a. Similarly, temporal characteristics are vital, as traffic volumes at specific times are affected by conditions at previous moments, as shown in Fig. 1b. In addition, cyclical variations in traffic, such as peak hours in the morning and evening followed by stable periods, as well as specific anomalies, lead to hidden correlations between periodicity and magnitude, phase in the frequency domain connected to future traffic conditions, as illustrated in Fig. 1b. These complex spatiotemporal characteristics significantly impact the accuracy and reliability of traffic predictions.

In this paper, we propose an innovative traffic prediction model called the Space-Time-Frequency Attention Network (STFAN) by integrating dynamic graph convolutional networks with conventional attention networks, which allows the model to explore spatial, temporal, and frequential characteristics inherent in traffic flow. Specifically, attention mechanisms are applied in both the time and frequency domains to capture the temporal features of traffic flow. In the time domain, the emphasis is placed on the evolving characteristics of local temporal dimensions, whereas in the frequency domain, the Fourier transform is utilized to examine frequency components, periodic features, and enduring patterns in traffic data. Incorporating these insights, the network seeks to achieve a thorough understanding of traffic patterns in space, time, and frequency dimensions, with the ultimate goal of enhancing the accuracy of traffic forecasts. The main contributions of this paper are as follows:

- We propose a model called the Space-time-Frequency Attention Network (STFAN) for traffic prediction. We incorporate frequency domain features of traffic flow into the network using spectral analysis. By integrating an attention mechanism, we deeply explore the hidden correlations of traffic features across space, time, and frequency dimensions, with the aim of improving the accuracy of traffic predictions at future time points.
- We examine cross-learning between traditional attention networks and GCN models. By incorporating the spatial/topological correlations of traffic flow, we map the traffic features to the GCN network for dynamic crossover through the attention mechanism, enabling us to further explore the spatial correlations of traffic features.
- We evaluate the performance of STFAN by conducting experiments on two real-world datasets. The results show that our STFAN model outperforms other models in terms of prediction accuracy, particularly for mid- and long-term predictions. In addition, we verify the effectiveness of STFAN through an ablation study, analyzing the impact of different modules on its predictive capability.

The remainder of this paper is organized as follows. In section “Problem statement”, we formally define the traffic forecasting problem. In section “Space-time-frequency attention networks”, we present the Space-Time-Frequency Attention Network (STFAN) for the prediction of traffic flow. In section “Experiments”, we carry out experiments on two real-world datasets to evaluate the performance of the proposed model, comparing it against several baseline models. Finally, we conclude the paper in section “Conclusion”.

## Related work

Researchers integrated spatial-temporal traffic data and employed graph neural networks to forecast flow. These approaches effectively capture and model the intricate dynamics of spatial-temporal dependencies in traffic. For example, advanced spatiotemporal traffic prediction models utilize Graph Neural Networks (GNNs) to gather information from adjacent nodes and create dynamic graphs using data-driven methods^23,24^. To capture the spatiotemporal dependencies of traffic data more comprehensively, Graph Convolutional Networks (GCNs) have also been applied, allowing the representation of spatiotemporal correlations in time series within the non-Euclidean structure of road networks^15,25,26^. However, numerous existing GCNs depend on static adjacency matrices to depict the spatial correlations in road networks. This approach fails to capture these networks’ dynamic nature of spatial dependencies accurately. With the emergence of attention mechanisms and their widespread application in various fields, traffic flow forecasting has also benefited significantly from the self-attention mechanism of the model, which excels at capturing interdependencies within input sequences^17–19^. Guo et al. introduced the Attention-based Spatio-Temporal Graph Convolutional Network (ASTGCN), which leverages attention mechanisms to enhance the model’s ability to capture and project spatiotemporal dependencies^27^. These advancements highlight the significance and potential of incorporating deep learning with spatial and temporal dynamics in traffic analysis. Building on this foundation, Xu et al. developed the Spatio-Temporal Transformer Network (STTN), which accounts for both spatial correlations and extended temporal sequences, significantly improving the accuracy of long-term traffic flow predictions^17^. Fang et al. proposed a Locality-aware spatiotemporal joint Transformer (Lastjormer), in which spatiotemporal joint attention is incorporated into the Transformer architecture to extract the correlation between sensors. This is achieved by utilizing dot-product self-attention over multiple time slots^28^. Recently, Feng and Tassiulas introduced an adaptive graph spatial-temporal transformer network designed to accommodate cross-temporal influences, thereby improving the effectiveness of traffic prediction^22^. These models have enhanced the practical application of traffic flow prediction and brought forth novel methods for practical use.

In recent years, some research on time-frequency attention analysis has appeared. Fang et al. proposed an effective model called Temporal-Frequency Masked Auto-Encoder (TFMAE). This model leverages two transformer-based self-encoders, incorporating a window-based temporal masking strategy and an amplitude-based frequency masking strategy, to acquire knowledge about anomaly-free bias and extract normal information for reconstructing anomalies^29^. Yang et al. introduced a novel Adaptive Time-Frequency Network (ATFN), an end-to-end hybrid model that combines deep learning networks with frequency patterns. This model utilizes an augmented sequence-to-sequence model to learn the trend characteristics of complex non-stationary time series. It captures the dynamics and intricate periodic patterns of time series data through frequency domain blocks and integrates trend and periodic features using a fully connected neural network for final prediction^30^. Fang et al. the discrete wavelet transform is leveraged to obtain the low- and high-frequency components of traffic sequences, and a dual-channel encoder is elaborately designed to capture the spatio-temporal dependencies under long accurately- and short-term schemas of the low- and high-frequency components^31^. However, the complicated hidden relationship between traffic data in the frequency domain and spatial-temporal has always been an investigation worthy of research, especially the effect of the complex interrelation between amplitude and phase in the frequency domain and spatial-temporal on the state of traffic at future moments.

**Fig. 2:**
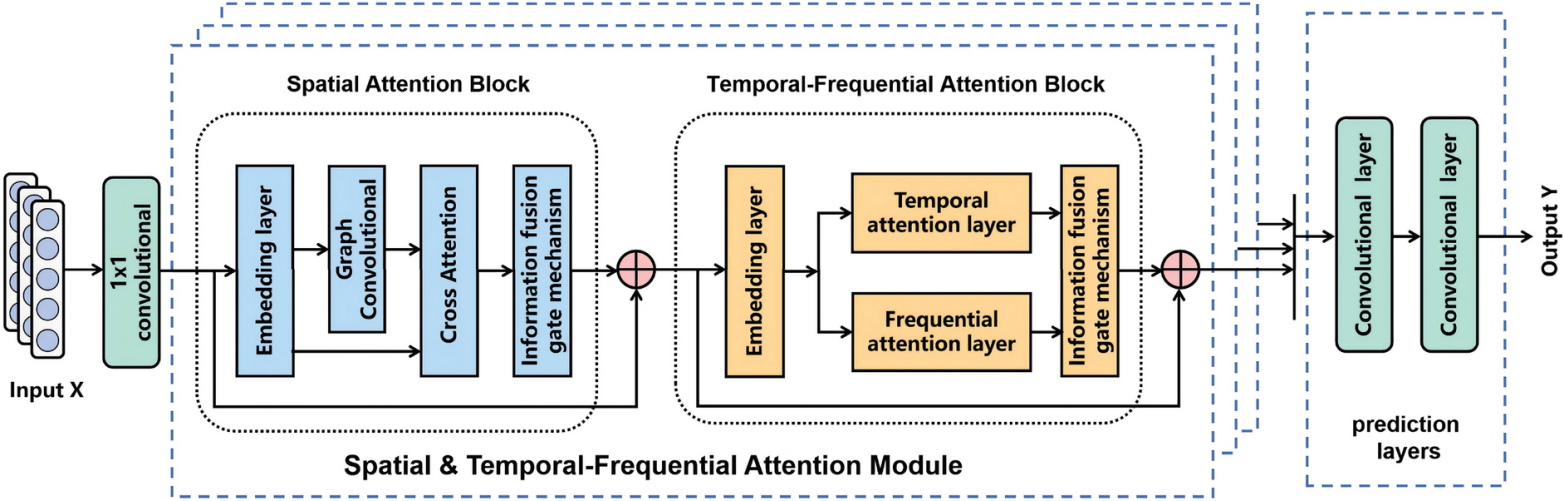
The overall architecture of the STFAN framework. The framework consists of two main components: the stacked spatial and temporal-frequential attention modules and a prediction layer. Each spatial and temporal-frequential attention module consists of a spatial attention block and a temporal-frequential attention block.

## Problem statement

In this section, we formally define the problem of traffic flow prediction.

**Traffic network**: The traffic network is defined as an undirected graph $\mathscr {G} =(\mathscr {N},\mathscr {E}, {A})$, where $\mathscr {N}$ denotes a set of *N* nodes, $\mathscr {E}$ denotes the set of edges, and *A* denotes the adjacency matrix. Assuming that each detector in the network $\mathscr {G}$ collects traffic data at a uniform sampling frequency *f*, each node generates a feature vector of length *f* at each time slice.

**Traffic tensor**: We denote $x_{t}^{i}\in {\mathbb {R}}$ the features of node *i* at moment *t*, and $X_{t}=[x_{t}^{1},x_{t}^{2},\dots ,x_{t}^{N} ]\in {\mathbb {R}}^{N}$ denote the set of all nodes in the dimensions at moment *t*. For a given *N* nodes, the observed *M* historical traffic data are $X=[X_{t-M+1},X_{t-M+2},\dots ,X_{t}]\in \mathbb {R}^{N\times M}$.

**Traffic flow prediction**: We adopt a multi-step prediction strategy for future traffic data based on long-term time dependence^17^. Given *M* historical traffic conditions $[X_{t-M+1}, X_{t-M+2},\dots , X_{t}]$ observed by the *N* sensors and a traffic network $\mathscr {G}$, through the network to output the prediction data $Y=[y_{t+1},y_{t+2},\dots ,y_{t+T}]\in \mathbb {R}^{N\times T}$ sequentially to achieve the process of multiple input to multiple output, as shown in Eq. (1).

1 $$\begin{aligned} \begin{aligned} {[}y_{t+1},\dots ,y_{t+T}{]}=\mathscr {F}([X_{t-M+1},\dots ,X_{t}];G) \end{aligned} \end{aligned}$$

## Space-time-frequency attention networks

In this section, we provide a comprehensive introduction to the STFAN model. This model has the ability to dynamically capture the spatial characteristics of the traffic data while simultaneously merging the time and frequency domains to collectively capture the spatiotemporal dependencies.

### Model architecture

The primary architecture of the model is shown in Fig. 2, consisting of stacked spatial and temporal-frequential modules and a prediction layer. Each spatial and temporal-frequential module includes a spatial attention block and a temporal-frequential attention block, with the latter focused on learning temporal dependencies of traffic data in both the time and frequency domains. These modules collaborate to capture concealed correlations in traffic characteristics by modeling dynamic temporal and frequential patterns, while also considering the spatial topology of the road network. The prediction layer then applies two classical $1\times 1$ convolutional layers to perform multi-step forecasting based on the spatiotemporal features extracted from the spatial and temporal-frequential modules.

#### Spatial and temporal-frequential module

The model explores the dependencies of traffic features by constructing multiple stacked spatial and temporal-frequential modules, as shown in Fig. 2. These modules, combined with attention mechanisms, aim to reveal the fundamental relationships of traffic characteristics across space, time, and frequency dimensions. Furthermore, by stacking these modules, the model extracts deep spatiotemporal features, enabling the creation of a robust and comprehensive prediction framework.

Initially, the input matrix $X \in \mathbb {R}^{N \times M}$ is transformed through a $1\times 1$ convolutional layer to obtain $X^S \in \mathbb {R}^{N \times M \times d}$, where *d* denotes the number of embedding channels. Subsequently, the matrix is fed into a Spatial and Temporal-Frequential (STF) module for training. For the *i*-th STF module, the input consists of the traffic features extracted from the *N* nodes at time steps $[t-M+1, \ldots , t]$ by the previous module $i-1$, denoted as $X_i^S \in \mathbb {R}^{N \times M \times d}$. The features $X_i^S$ are then dynamically fused with the graph adjacency matrix *A* and jointly input into the spatial attention block of the STF module for dynamic learning of spatial features, resulting in the spatial feature matrix $Y_i^S$, as illustrated in Eq. (2).

2 $$\begin{aligned} (X_i^{S},A)\overset{S}{\rightarrow }\ Y_i^{S} \end{aligned}$$

$Y_i^S$ is combined with $X_i^S$ to generate the input $X^{\mathscr {T}}_i=X_i^S+Y_i^S$, and then $X_i^{\mathscr {T}}$ passes through a temporal-frequential attention block to produce the temporal feature $Y_i^{\mathscr {T}}$, as in Eq. (3). Finally, the spatial input feature tensor $X_{i+1}^{S}$ of the $i+1$th module by the formula Eq. (4)

3 $$\begin{aligned} & (X_i^{\mathscr {T}})\overset{\mathscr {T}}{\rightarrow } Y_i^{\mathscr {T}} \end{aligned}$$

4 $$\begin{aligned} & X_{i+1}^S=X_i^{\mathscr {T}}+Y_i^{\mathscr {T}} \end{aligned}$$

#### Prediction layer

The predictive layer employs two classic convolutional layers to forecast the output features of the spatial and temporal-frequential module. Specifically, the multi-step prediction of the future traffic conditions for *N* nodes across *T* time steps, denoted by $Y \in \mathbb {R}^{N \times T}$, is accomplished as follows:

5 $$\begin{aligned} Y=Conv(Conv(Y^{\mathscr {T}})) \end{aligned}$$

The mean absolute loss is adopted to train the model.

6 $$\begin{aligned} L=||Y-Y^{gt}|| \end{aligned}$$

where $Y^{gt}$ is the ground-truth traffic features.

**Fig. 3:**
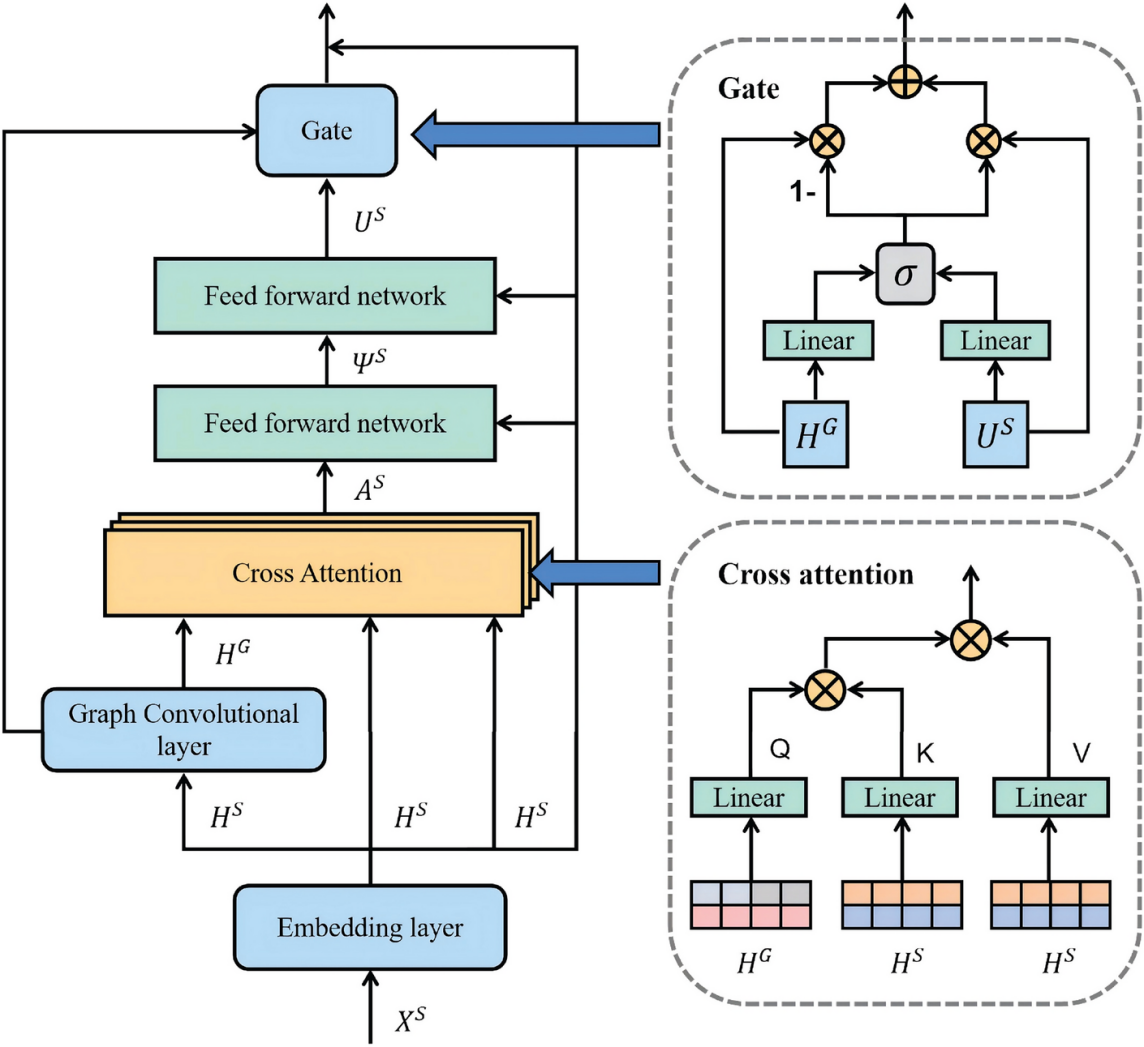
The architecture of a spatial attention block. It consists of four key components: a position embedding layer, a graph convolutional layer, a cross attention layer, and a gate mechanism.

### Spatial attention block

This section presents the spatial attention block of the STFAN model. The block comprises an embedding layer, a graph convolution layer, a cross-attention layer, and an information fusion gate mechanism. The detailed architecture is illustrated in Fig. 3. Given the intricate connections and dynamic changes among the nodes of the spatial road network, this study incorporates the Graph Convolutional Network (GCN) to capture and learn the spatial correlation of the road network. Additionally, a spatial attention layer is constructed to project traffic features in the road network using the attention mechanism, enabling a deeper exploration of potential connections among traffic features in space. The attention mechanism is employed to focus on key spatial features and is combined with the graph structure analysis capability of the GCN to achieve a more comprehensive understanding of traffic states. A detailed description of each structure in the module is provided below.

#### Embedding layer

In this layer, the positional information of the feature vectors is integrated into the input sequence. There are two types of positional information: one is the spatial position in the traffic network topology, and the other is the temporal position embedded within the time series. Specifically, the embedding matrix for the spatial position denoted as $D^S$, is constructed and formulated in $\mathbb {R}^{N \times N}$. Temporal position is represented by the matrix $D^{\mathscr {T}}$, also in $\mathbb {R}^{M \times M}$. Following the methodology of Xu et al.^17^, $D^S$ is initialized using the graph adjacency matrix to explore the spatial dependency among network nodes; $D^{\mathscr {T}}$ is initialized using a one-hot time encoding to account for temporal dependencies. Subsequently, $D^S$ and $D^{\mathscr {T}}$ are flattened along the time and space dimensions, resulting in $\hat{D}^S$ in $\mathbb {R}^{M \times N \times N}$ and $\hat{D}^{\mathscr {T}}$ in $\mathbb {R}^{M \times N \times M}$, respectively. Then, by concatenating $\hat{D}^S$ with $X^S$, the final embedding $H^S$ is obtained as $F_t([X^S, \hat{D}^S])$ in $\mathbb {R}^{M \times N \times d}$. Here, $F_t$ represents a 1x1 convolutional layer that transforms the combined features of each node at each time step into a *d*-dimensional vector.

#### Graph convolutional layer

The module utilizes graph convolution based on Chebyshev polynomial approximations to acquire the structure-aware node features and subsequently capture the spatial dependencies present in the transportation network topology^32^. By combining data from nearby nodes according to specified graphs and learning weights, the nodes’ features are generated. The normalized Laplacian matrix is defined as follows given the adjacency matrix A’s degree matrix D: $L = I_{n}-D^{-1/2}AD^{-1/2}$. Furthermore, $\widetilde{L} = 2L/\lambda _{max} -I_{n}$ is the scaled Laplacian matrix of Chebyshev polynomials , where $\lambda _{max}$ is the maximum eigenvalue of *L*. The final structure-aware node features are obtained by approximating the graph convolution with *k*th order Chebyshev polynomials. The *j*th channel of $H^G$ is learned as follows:

7 $$\begin{aligned} H^{G}_{:,j}=\sum ^{d}_{i=1}\sum _{k=0}^{K}{\theta _{ij,k}T_{k}(\hat{L})H_{:,i}^{S} } \end{aligned}$$

where $H_{:,j}^{G}$ is the *i*th channel of the node feature and $\theta _{ij,k}$ is the learnable parameter.

#### Cross attention layer

This layer combines traffic features with graph convolutional networks using an attention mechanism to dynamically learn the spatial properties of traffic features. Firstly, the output feature $H^S$ of each node is projected into a high-dimensional subspace using a feed-forward network after passing through the embedding layer. This generates a query subspace $Q^S$ and a key subspace $K^S$. Then, the features $H^G$ generated by passing the input features $H^S$ through the graph convolutional network are also projected into the high-dimensional subspace using the feed-forward network to generate the value subspace $V^G$. These can be computed using the following equation:

8 $$\begin{aligned} \begin{aligned} Q^S&=H^SW^S_q \\ K^S&=H^SW^S_k \\ V^G&=H^GW^G_v \end{aligned} \end{aligned}$$

Here, $W^S_q \in \mathbb {R}^{N\times d}$,$W^S_k \in \mathbb {R}^{N\times d}$, and $W^G_v \in \mathbb {R}^{N\times d}$ are the weight matrices, respectively. The dependence $Z^S$ of the traffic features on the dynamic space is then computed by scaled dot-product and normalization operations.

9 $$\begin{aligned} \begin{aligned} Z^S=\text {softmax}(Q^S(K^S)^T/\sqrt{d}) \end{aligned} \end{aligned}$$

where $Z^S$ represents the dynamic dependency matrix between nodes. softmax() represents the transformation of the matrix into a probability distribution. $\sqrt{d}$ represents the scaling factor used to prevent saturation of softmax when the gradient is extremely small. Subsequently, the dependencies $Z^S$ between traffic features are projected onto the spatial features computed by the graph convolutional network. The traffic features are then combined with the spatial features, resulting in a new feature $A^S$ derived using the following formula:

10 $$\begin{aligned} \begin{aligned} A^S=Z^S\times V^G \end{aligned} \end{aligned}$$

The features $A^S$ are then fed into a multilayer feed-forward neural network with a nonlinear activation function for training, thus exploiting the dependencies of the features in various hidden spaces.

11 $$\begin{aligned} \begin{aligned} U^S=ReLU(ReLU(\Psi ^{S}W^{S}_{0})W^{S}_1)W^{S}_2 \end{aligned} \end{aligned}$$

where ReLU() represents the nonlinear activation function, $\Psi ^{S}=H^S+A^S$ denotes the residual connection after stabilization training, and $W_{0}^{S}$, $W_{1}^{S}$, and $W_{2}^{S}$ refer to the weight matrices. To enhance the prediction of node features, the generated data is merged with the initial data. This merged dataset is subsequently fed into a multilayer feed-forward neural network that incorporates nonlinear activation functions. This enables the neural network to effectively identify the complex interrelationships among different channels, thereby enhancing its prediction capability.

#### Information fusion gate mechanism

To integrate the spatial features learned from the graph convolution and attention layers, we employ the Gate mechanism. This involves learning a gate as a means to fuse the features:

12 $$\begin{aligned} \begin{aligned} g=\text {sigmoid}(f_S(\Omega ^S)+f_G(H^G)+b) \end{aligned} \end{aligned}$$

where sigmoid() maps input values into the interval (0, 1) and is typically used in binary classification problems to represent probabilities. $f_S()$, $f_G()$ denotes the fully connected layer and *b* is the bias term. The final output of the spatial transformer is:

13 $$\begin{aligned} \begin{aligned} Y^S=g\Omega ^S+(1-g)H^G \end{aligned} \end{aligned}$$

### Temporal-frequential attention block

**Fig. 4:**
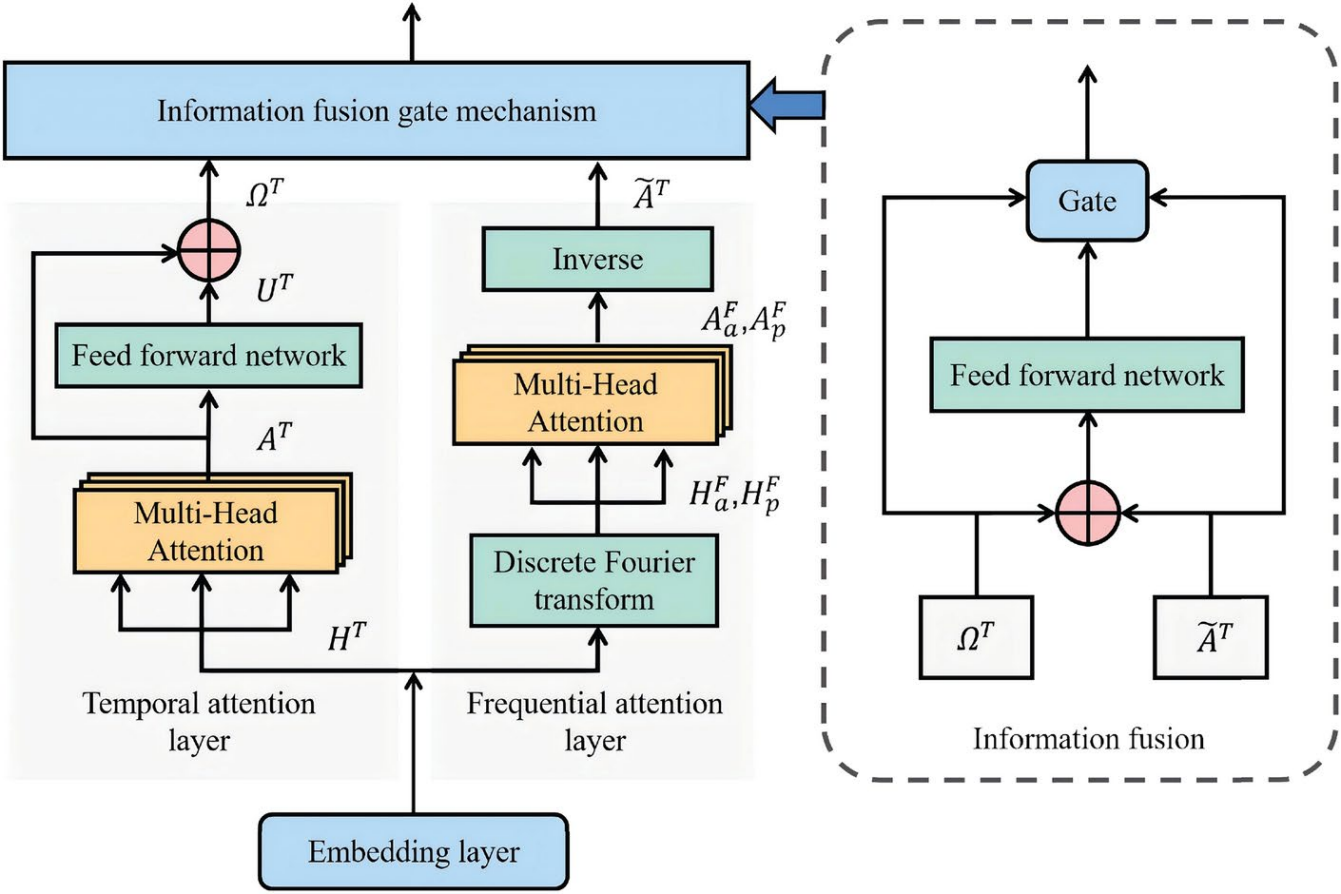
The architecture of the temporal-frequential attention block. It consists of a position embedding layer, a temporal attention layer, a frequential layer, and an information fusion gate mechanism.

The temporal-frequential attention block is designed to analyze the underlying connections within data over time. This block effectively captures and learns the hidden correlations of traffic features by dividing the flow into two dimensions: the time domain and the frequency domain. The specific architecture of the module is illustrated in Fig. 4.

#### Temporal attention layer

Similar to the spatial attention block, the input $H^{\mathscr {T}} = G_t([X^{\mathscr {T}}, D^{\mathscr {T}} ]) \in \mathbb {R}^{M\times d}$ of the module is obtained from the concatenation of the input features $X^{\mathscr {T}} = X^S + Y^S\in \mathbb {R}^{M \times N \times d}$ and temporal embedding $D^{\mathscr {T}}$, where $G_t$ is a $1 \times 1$ convolutional layer. Then $H^{\mathscr {T}}$ is mapped to the high-dimensional subspace to generate the query subspace $Q^{\mathscr {T}}$, key subspace $K^{\mathscr {T}}$ and value subspace $V^{\mathscr {T}}$. By focusing on key time points in the sequence through the attention mechanism, the hidden associations inherent in the sequence data are captured, such as Eq. (14):

14 $$\begin{aligned} \begin{aligned} Z^{\mathscr {T}}&=\text {softmax}(Q^{\mathscr {T}}(K^{\mathscr {T}})^T/\sqrt{d}) \\ A^{\mathscr {T}}&=Z^{\mathscr {T}}\times V^{\mathscr {T}} \end{aligned} \end{aligned}$$

The temporal attention layer learns the hidden correlations of sequences at different time intervals. Since a single time series, while reflecting specific information at a particular moment in time, fails to reveal the complex correlations that may exist between different moments. The hidden relationship between the time points has been further learned through the multiple attention mechanism to obtain the feature $A^{\mathscr {T}}$. To explore the dependencies of the time domain data more deeply, different data are inputted into a multilayer feed-forward neural network with a nonlinear activation function for training, as in Eq. (15).

15 $$\begin{aligned} \begin{aligned} U^{\mathscr {T}}=ReLu(ReLu(A^{\mathscr {T}}W^{\mathscr {T}}_{0})W^{\mathscr {T}}_1)W^{\mathscr {T}}_2 \end{aligned} \end{aligned}$$

where ReLu() represents the nonlinear activation function, and $W_{0}^{\mathscr {T}}$, $W_{1}^{\mathscr {T}}$, and $W_{2}^{\mathscr {T}}$ refer to the weight matrices. Then $U^{\mathscr {T}}$ and $A^{\mathscr {T}}$ are merged to generate $\Omega ^{\mathscr {T}}$, which is passed to the lower level to continue learning.

#### Frequential attention layer

The frequential attention layer is primarily utilized to learn the frequency domain features of time series data. Considering that traffic data is sampled at discrete intervals and shows periodic patterns over time, the discrete Fourier transform (DFT) is useful for examining the traffic data in the frequency domain^33^. This analysis helps to learn the distribution pattern of the traffic data at different frequencies and uncover the underlying connections within the frequency domain. Additionally, it allows for the exploration and comprehension of the dynamic patterns and periodic laws present in the data. The new frequency feature $H^{\mathscr {F}}$ can be obtained by performing a Fourier transform of the feature $H^{\mathscr {T}}$ through Eq. (16).

16 $$\begin{aligned} \begin{aligned} H^{\mathscr {F}}(k) = \sum _{n=1}^{M} H^{\mathscr {T}}(n) \cdot e^{-j\frac{2\pi }{M}k(n-1)} \end{aligned} \end{aligned}$$

Where $H^{\mathscr {F}}(k)$ denotes the *k*th component in the frequency domain. $H^{\mathscr {T}}(n)$ is the *n*th sampling point in the time domain. *M* is the total length of the sequence, *j* is the imaginary unit. In practice, $H^{\mathscr {T}}(n)$ is a real signal, which means that the imaginary part is 0. In this case, the Eq. (16) can be expanded as follows:

17 $$\begin{aligned} \begin{aligned} H^{\mathscr {F}}(k)=\sum _{n=1}^{M}H^{\mathscr {T}}(n) \left( \cos {2\pi k\frac{n}{M}}-j\sin {2\pi k\frac{n}{M}}\right) \end{aligned} \end{aligned}$$

Extract the amplitude feature $\hat{H}^{\mathscr {F}}_{a}=|H^{\mathscr {F}}|$ and phase $\hat{H}^{\mathscr {F}}_{p}={2\pi k\frac{n}{M}}$, map them to a high-dimensional subspace to generate the query subspace $Q^{\mathscr {F}}_{a}$,$Q^{\mathscr {F}}_{p}$, the key subspace $K^{\mathscr {F}}_{a}$,$K^{\mathscr {F}}_{p}$ and the value subspace $V^{\mathscr {F}}_{a}$,$V^{\mathscr {F}}_{p}$. Then, utilize the attentional mechanism as described in Eq. (18) to learn the hidden correlations in the sequence and obtain the new feature $A^{\mathscr {F}}_a$, $A^{\mathscr {F}}_p$, and reduce the time-domain signals $\tilde{A}^{\mathscr {T}}$ by Fourier inverse transforms.

18 $$\begin{aligned} \begin{aligned} A^{\mathscr {F}}_a&=(\text {softmax}(Q^{\mathscr {F}}_a(K^{\mathscr {F}}_a)^T/\sqrt{d}))\times V^{\mathscr {F}}_a \\ A^{\mathscr {F}}_p&=(\text {softmax}(Q^{\mathscr {F}}_p(K^{\mathscr {F}}_p)^T/\sqrt{d}))\times V^{\mathscr {F}}_p \\ \tilde{A}^{\mathscr {T}}(n)&=\frac{1}{2\pi }\int _{-\infty }^{+\infty } A^{\mathscr {F}}(k)e^{j(2\pi kn/N + A_p)}dk \end{aligned} \end{aligned}$$

#### Information fusion

To fuse the features and explore the hidden dependencies of the different dimensional features, it is necessary first to fuse the time-domain feature $\Omega ^{\mathscr {T}}$ and the frequency-domain feature $\tilde{A}^{\mathscr {T}}$ to generate feature $\Phi ^{\mathscr {T}}=\Omega ^{\mathscr {T}}+\tilde{A}^{\mathscr {T}}$. Then feature $\Phi ^{\mathscr {T}}$ is introduced into the multilayer feed-forward neural network with a nonlinear activation function for training. The hidden correlations of the fused features are explored in depth. Finally, the fused feature $\Phi ^{\mathscr {T}}$ is input into the gate mechanism together with the original features to get the output result $Y^{\mathscr {T}}$.

19 $$\begin{aligned} \begin{aligned} g&=\text {sigmoid}(f_{\mathscr {T}}(\Omega ^{\mathscr {T}})+f_{\mathscr {F}}(\tilde{A}^{\mathscr {T}})+b) \\ Y^{\mathscr {T}}&=g\Omega ^{\mathscr {T}}+(1-g)(\tilde{A}^{\mathscr {T}}) \end{aligned} \end{aligned}$$

## Experiments

In this section, we present the experimental results of our proposed STFAN model for traffic flow prediction. This includes a comparative analysis of our model’s performance against several recent benchmark models, as well as a detailed ablation experiment to assess the contributions of the spatial, temporal, and frequential layers in the network architecture.

### Datasets

The dataset is derived from the Caltrans Performance Measurement System (PeMS), which contains more than 44,681 detectors that cover highways in major metropolitan areas of California, USA. Every 5-min traffic data is aggregated into a one-time slice of data. Thus, each hour contains 12-time slices of traffic information, while each day contains 288 time slices. Each detector corresponds to each node in the graph network. The datasets used in this paper are PeMS04 and PeMS08, which include three traffic characteristics: flow, speed, and occupancy. Table 1 provides the specifics of the dataset.

**Table 1:** Details of the datasets.

Datasets	Nodes	Edges	Time steps	Time range
PeMS04	307	340	16,992	2018/01/01–2018/02/28
PeMS08	170	295	17,856	2016/07/01–2016/08/31

1. PeMS04: This dataset covers 3,848 traffic detectors on 29 freeways in the San Francisco Bay Area, providing traffic data from January 1, 2018, to February 28, 2018.
2. PeMS08: This dataset contains traffic data for the city of San Bernardino for the period July 1 to August 31, 2016, covering 1,979 traffic detectors on eight roadways.

In a comparative study of the STFAN alongside other models, the benchmark models chosen from recent years are:

- XGBoost (2016)^34^: A widely utilized method in regression tasks is the classic gradient-boosting tree-based approach.
- FC-LSTM (2015)^35^: The Long Short-Term Memory Network utilizes fully connected layers to enhance the information of the original data.
- DCRNN (2018)^15^: The Diffusion Convolution Recurrent Neural Network combines a bi-directional random walk on a distance-based graph with a Gated Recurrent Unit (GRU) in an encoder-decoder manner.
- STGCN (2018)^25^: Spatio-temporal Graph Convolutional Networks employ graph convolution and causal convolution to acquire spatial and temporal dependencies.
- Graph WaveNet (2019)^36^: A framework integrates an adaptive adjacency matrix into graph convolution with 1D dilated convolution.
- STSGCN (2020)^37^: The Spatial-Temporal Synchronous Graph Convolutional Network utilizes a localized spatio-temporal subgraph module to independently model localized correlations.
- AGCRN (2020)^38^: The Adaptive Graph Convolutional Recurrent Network decomposes the adjacency matrix and parameters of the graph convolution layer.
- STFGNN (2021)^39^: The Spatial-Temporal Fusion Graph Neural Network constructs a temporal graph by learning similarities between time series.
- K-STTN (2023)^40^: The model is a knowledge-induced spatiotemporal transformer network designed to simultaneously forecast traffic flow and speed by incorporating Greenshields’ traffic model to capture spatiotemporal dependencies.
- ST-AE (2023)^41^: Spatio-Temporal AutoEncoder is an autoencoder specially designed to learn the intrinsic patterns from traffic flow data and encode the current traffic flow information into a low-dimensional representation.

**Table 2:** The results of traffic flow prediction with respect to different models. Significant values are in bold.

Dataset	Models	15 min			30 min			60 min		
		MAE	MAPE	RMSE	MAE	MAPE	RMSE	MAE	MAPE	RMSE
PeMS04	XGBoost	22.816	0.157	33.715	26.780	0.189	39.012	35.678	0.274	50.372
	FC-LSTM	22.793	0.186	37.699	22.870	0.186	37.813	23.184	0.185	38.176
	DCRNN	19.653	0.152	31.297	21.808	0.168	34.111	26.200	0.184	39.919
	STGCN	19.704	0.148	31.150	20.707	0.153	32.862	22.140	0.169	34.995
	Graph WaveNet	18.753	0.141	29.804	20.406	0.158	31.918	23.218	0.194	35.414
	STSGCN	19.687	0.131	31.052	21.180	0.139	33.316	24.613	0.161	38.135
	AGCRN	19.137	0.128	30.596	20.395	0.137	32.428	23.106	0.158	36.218
	STFGNN	18.723	0.123	29.983	19.756	0.129	31.697	21.648	0.140	34.342
	K-STTN	18.314	0.136	29.484	19.341	0.139	31.012	21.052	0.151	32.761
	ST-AE	**18.262**	0.133	**29.194**	19.523	0.145	31.113	21.246	0.175	33.421
	STFAN	18.372	**0.119**	29.506	**18.938**	**0.122**	**30.446**	**19.793**	**0.127**	**31.857**
PeMS08	XGBoost	16.976	0.110	26.114	19.954	0.132	30.375	26.660	0.188	39.285
	FC-LSTM	21.688	0.139	35.221	21.959	0.139	35.381	23.024	0.145	37.238
	DCRNN	15.049	0.101	23.313	16.474	0.109	25.815	18.848	0.123	29.632
	STGCN	15.162	0.106	23.269	16.404	0.111	25.374	18.670	0.121	28.679
	Graph WaveNet	14.922	0.102	23.342	16.688	0.107	26.101	19.288	0.128	29.900
	STSGCN	15.727	0.101	24.234	17.003	0.108	26.370	19.699	0.124	30.337
	AGCRN	15.108	0.096	23.654	16.093	0.103	25.419	18.165	0.117	28.698
	STFGNN	15.409	0.099	23.928	16.767	0.107	26.308	19.429	0.122	30.250
	K-STTN	14.226	0.095	22.374	15.012	0.098	24.138	16.752	0.113	25.630
	ST-AE	**14.171**	0.094	22.398	15.250	0.099	24.353	17.243	0.115	27.349
	STFAN	14.203	**0.093**	**22.161**	**14.720**	**0.095**	**23.133**	**15.556**	**0.098**	**24.567**

### Experimental settings

We implement the STFAN model based on the PyTorch deep learning framework and use Python 3.6 as the runtime environment. The experimental setup is as follows: The PeMS04 dataset and PeMS08 dataset contain traffic data for 59 and 62 days, respectively. We divide the training set, validation set, and test set in a ratio of 6:2:2. The time step of the input is set to 12, and the prediction step is also set to 12. In the model training phase, the batch size is set to 16, the epoch is set to 120, the number of heads is set to 8, and the learning rate is set to 0.001. In the experiments on the dataset, the number of stacked spatial-temporal transformer modules is set to 3, the parameter k of the Chebyshev polynomial is set to 3, and the number of heads of the multiheaded attention mechanism is set to 3. The optimizer for model training is AdamW. The mean square error (MSE) between the true and predicted values is used as the loss function.

In this study, we employ widely recognized metrics to assess the model’s predictive accuracy, namely (i) Mean Absolute Error (MAE), (ii) Mean Absolute Percentage Error (MAPE), and (iii) Root Mean Squared Error (RMSE), computed by the following Eq. (20):

20 $$\begin{aligned} \begin{aligned} MAE&=\frac{1}{n}\sum ^{n}_{i=1}|Y_i-\hat{Y_i}| \\ MAPE&=\frac{1}{n}\sum _{i=1}^{n}|\frac{Y_i-\hat{Y_i}}{Y_i}| \\ RMSE&=\sqrt{\frac{1}{n}\sum _{i=1}^{n}(Y_i-\hat{Y_i})^2} \end{aligned} \end{aligned}$$

**Fig. 5:**
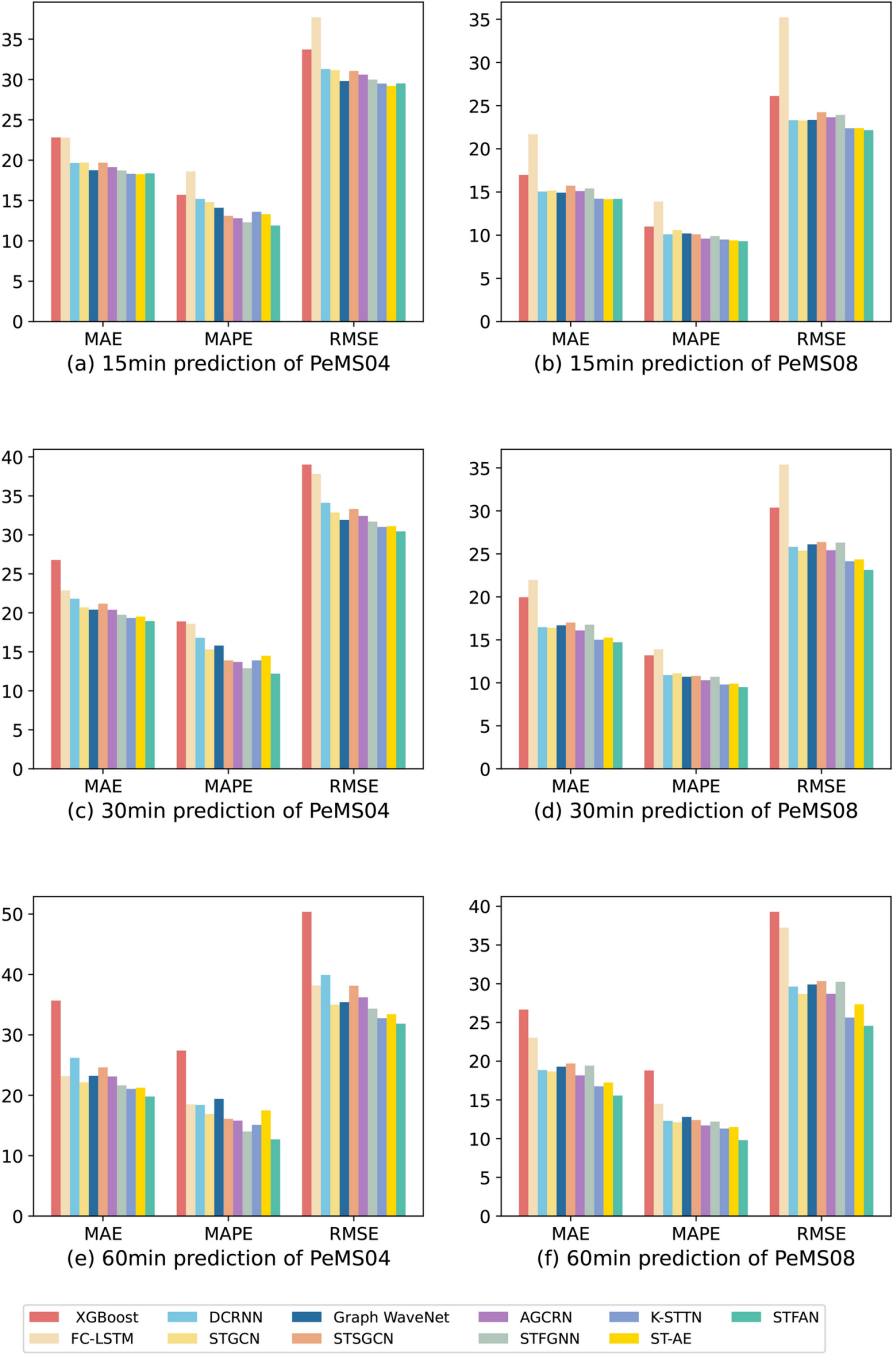
Comparison of traffic flow prediction metrics between the proposed STFAN model and benchmark methods on the PeMS04 and PeMS08 datasets for 15-min, 30-min, and 60-min prediction intervals.

### Comparative analysis

The traffic flow data from the PeMS04 and PeMS08 datasets are used as inputs for the proposed STFAN model to predict traffic flows at future time points. These datasets are also used as inputs for the various benchmark models mentioned earlier to generate their predictions. The accuracy of the predictions made by the STFAN model is then compared with those of the benchmark models. Table 2 presents a comparison of the actual and predicted values across different evaluation metrics for a one-hour prediction window with respect to each model.

A comparative analysis of the prediction results from the models listed in Table 2 on the PeMS04 and PeMS08 datasets reveals that the STFAN model demonstrates superior accuracy in forecasting traffic flow beyond a 30-min horizon. Specifically, for predictions spanning 30 min, the STFAN model consistently outperforms all benchmark models in both datasets. In the PeMS04 dataset, the STFAN model achieves a reduction of 1.02 times in MAE, 1.05 times in MAPE, and 1.02 times in RMSE compared to the best-performing baseline model. Similarly, in the PeMS08 dataset, the STFAN model reduces MAE, MAPE, and RMSE by 1.02, 1.03, and 1.04 times, respectively.

In the case of 60-min forecasting horizons, the STFAN model continues to outperform the baseline, showing a 1.06 times decrease in MAE, 1.1 times in MAPE, and 1.03 times in RMSE on the PeMS04 dataset, and 1.08 times, 1.15 times, and 1.04 times reduction on the PeMS08 dataset respectively.

For short-term traffic flow predictions of under 15 min, the STFAN model exhibits a relatively better performance in MAPE, with reductions of 1.03 times and 1.01 times on the PeMS04 and PeMS08 datasets respectively. However, the MAE and RMSE values show a slight increase of 1.01 times in the PeMS04 dataset, 1.002 times in the PeMS08 dataset for MAE, and a slight decrease of 1.009 times for RMSE on the PeMS08 dataset. The less pronounced short-term prediction performance may be attributed to the difficulty in accurately expressing the period and amplitude of traffic flow in the frequency domain within such a short time frame, as the dynamics of traffic flow may not form distinct periodic patterns within a brief period such as 15 min. This is compounded by the incompleteness of short-term traffic data, leading to potential increases in errors during the transformation from the time domain to the frequency domain. To further visualize these results, we have created bar charts for the aforementioned metrics, which clearly illustrate the distribution of the new model in relation to each evaluation criterion, as shown in Fig. 5.

**Table 3:** Results of the ablation study with respect to the PeMS08 dataset.

Modules	15 min			30 min			60 min		
	MAE	MAPE	RMSE	MAE	MAPE	RMSE	MAE	MAPE	RMSE
S+T	14.411	0.097	22.680	15.203	0.099	23.845	15.981	0.106	25.271
S+F	15.297	0.102	23.563	15.952	0.104	24.693	17.065	0.109	26.516
T+F	15.091	0.099	23.696	16.624	0.108	26.291	19.637	0.128	31.405
S+T+F	14.203	0.093	22.161	14.720	0.095	23.133	15.556	0.098	24.567

S+T: The model with Spatial attention block and Temporal attention layer. S+F: The model with Spatial attention block and Frequential attention layer. T+F: The model with the Temporal attention layer and Frequential attention layer. S+T+F: The model with all layers

### Ablation study

**Fig. 6:**
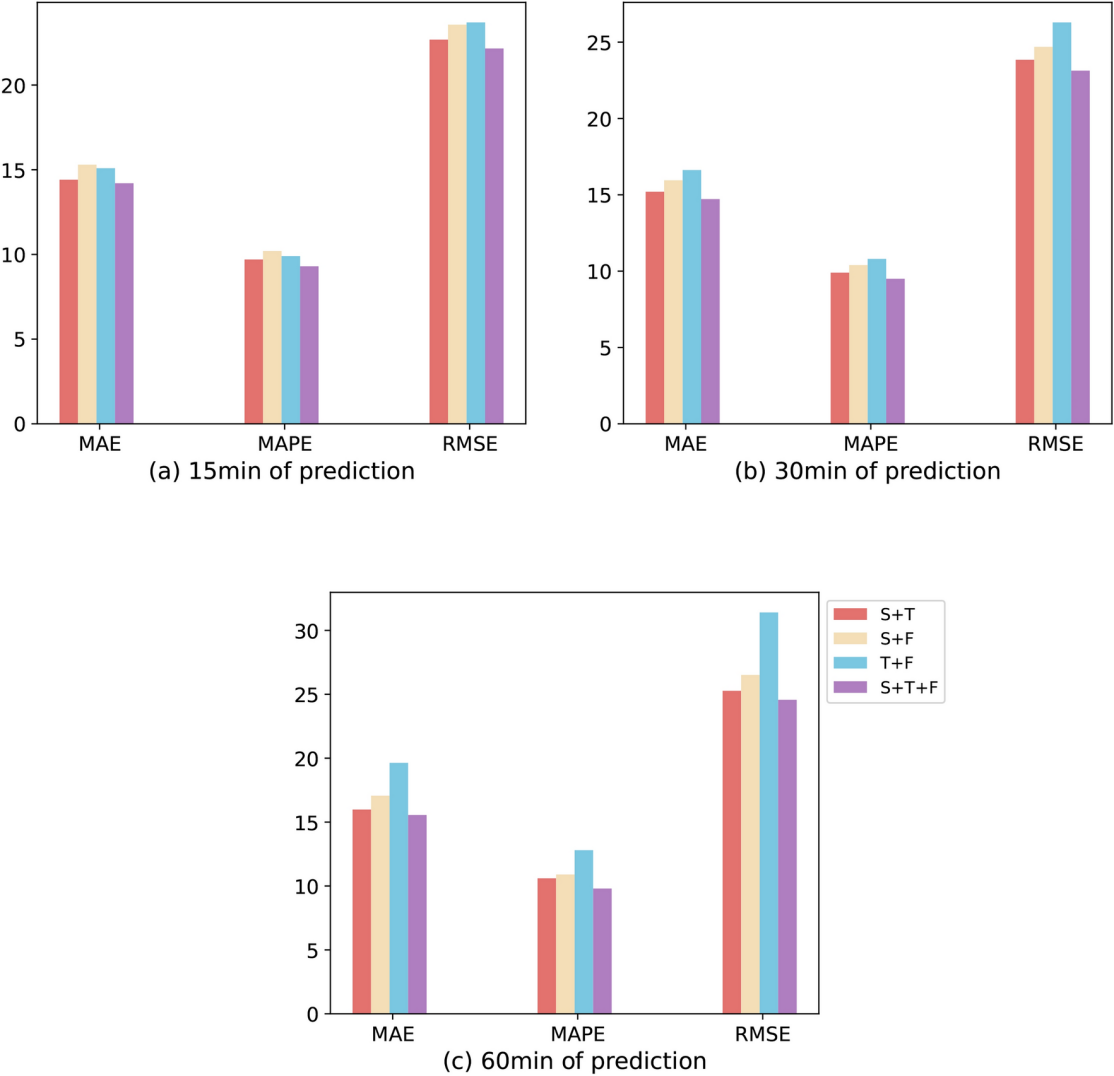
Results from the ablation study on the PeMS08 dataset, comparing STFAN with various modules removed, for prediction intervals of 15 min, 30 min, and 60 min.

To evaluate the impact of spatial, temporal, and frequential blocks on traffic learning, we conducted a predictive analysis through an ablation study. We sequentially eliminated spatial attention blocks (S), temporal attention layers (T), and frequential attention layers (F), and compared the predictions of the network for the next hour with those of the model with the complete architecture. Table 3 presents the final predictive results, which tested the network’s one-hour-ahead 15-min, 30-min, and 60-min predictive outcomes when different modules were removed, all in the PeMS08 dataset. Evaluation metrics for each time interval were calculated using the actual and predicted data of all nodes. To present these results more intuitively, we created bar charts to illustrate the distribution of each evaluation metric in Fig. 6. It can be observed from these charts that the absence of each module does indeed affect the accuracy of predictions for each time period. Specifically, the absence of the spatial attention block (T+F) increased the average prediction accuracy across different time periods by 1.05, 1.13, and 1.27, respectively; the absence of the frequential attention layer (S+T) affected the average prediction accuracy by 1.01, 1.02, and 1.02; and the absence of the temporal attention layer (S+F) increased the impact on average prediction accuracy by 1.06, 1.07, and 1.08. The ablation experiments further validate the influence of space, time, and frequency characteristics on the prediction of future traffic features, and also confirm the effectiveness of the STFAN model.

In addition, we provide an in-depth analysis of the Attention mechanisms and the number of their heads, with comparative experiments to evaluate the differences in predictive effectiveness of the different models. Specifically, we selected soft-attention and hard-attention mechanisms to compare them with the self-attention mechanism used in our model. For selecting heads in the self-attention, we tested 2 heads, 4 heads, and the 8 heads chosen for our model. By performing predictions at different periods on the PeMS08 dataset, the experimental results, as demonstrated in Table 4, show that the self-attention mechanism provides the best prediction results at all tested periods. The hard attention mechanism closely follows it. Furthermore, we observed that the prediction accuracy of the model increased as the number of attentional heads increased. These findings further validate the validity and reliability of our constructed model. To evaluate the impact of different hyperparameter settings on the performance of the model, we performed a systematic analysis focusing on two key parameters: learning rate and batch size. For this analysis, we tested combinations of learning rates (0.01, 0.001, and 0.0001) and batch sizes (16 and 32). After thoroughly comparing the prediction results and performing an in-depth analysis, we found that the model achieved optimal learning performance on the current dataset with a learning rate of 0.001 and a batch size of 16.

**Table 4:** The effect of different hyperparameter settings on prediction results with respect to the PeMS08 dataset.

Hyperparameter	Values	15 min			30 min			60 min		
		MAE	MAPE	RMSE	MAE	MAPE	RMSE	MAE	MAPE	RMSE
	2	30.243	0.151	47.142	27.066	0.145	41.649	24.350	0.144	37.435
Self-attention	4	15.324	0.191	23.186	15.953	0.193	24.258	16.901	0.192	25.920
	8	14.203	0.093	22.161	14.720	0.095	23.133	15.556	0.098	24.567
Soft-attention	8	15.665	0.101	24.288	16.122	0.103	25.102	16.957	0.109	26.535
Hard-attention	8	14.989	0.095	23.454	15.505	0.098	24.416	16.266	0.104	25.819
Learning rate	0.01	15.545	0.102	23.745	16.153	0.103	24.872	16.815	0.107	26.026
	0.001	14.203	0.093	22.161	14.720	0.095	23.133	15.556	0.098	24.567
	0.0001	14.975	0.096	23.244	15.676	0.099	24.447	16.270	0.104	25.473
Batch size	16	14.203	0.093	22.161	14.720	0.095	23.133	15.556	0.098	24.567
	32	14.826	0.096	22.914	15.231	0.098	24.015	15.869	0.102	25.377

## Conclusion

In this study, we have developed the Space-Time-Frequency Attention Network (STFAN) for the spatiotemporal prediction of traffic flow. By decomposing the temporal characteristics of traffic flow into both time and frequency domains, STFAN enables a more comprehensive analysis of traffic patterns. Furthermore, we integrated the spatial topology of the traffic network with an attention mechanism that learns hidden states across spatial, temporal, and frequency dimensions, allowing for in-depth modeling of traffic dynamics from past to future time points. This multidimensional approach enhances the model’s sensitivity to dynamic changes in spatiotemporal series data and improves its ability to identify and integrate features across different dimensions, offering a richer and more precise framework for traffic analysis. To evaluate the performance of our proposed model, we have conducted experiments on two real-world datasets by comparing them against several baseline models. The results have demonstrated that our framework outperforms these benchmarks, particularly in medium- to long-term predictions. This highlights the model’s ability to uncover intrinsic, information-rich hidden states, providing a new perspective for traffic flow prediction, especially in the frequency domain.

In this study, the Fourier transform was selected for frequency analysis due to its simplicity, interpretability, and computational efficiency, which makes it particularly well suited for preliminary exploratory analysis and processing large-scale datasets. The effectiveness of the Fourier transform in our approach has been demonstrated through a detailed comparative analysis with benchmark models. However, we acknowledge the diversity of frequency transformation methods, particularly the significant potential of the wavelet transform for frequency domain analysis in traffic data. Future work could explore the selection of appropriate wavelet bases and scaling functions, as these choices greatly influence the performance of the wavelet transform. Furthermore, investigating the compatibility between the temporal attention layer and the wavelet transform, specifically its impact on preserving temporal information, offers another promising direction. These multidimensional explorations could advance the field of traffic prediction and enhance the applicability of frequency-based methods to complex, nonstationary datasets.

## Data Availability

Traffic flow data that support the findings of this study have been provided in the Caltrans Performance Measurement System (PeMS) with the Web address http://pems.dot.ca.gov.
